# Evolving treatment patterns and improved outcomes in relapsed/refractory mantle cell lymphoma: a prospective cohort study

**DOI:** 10.1038/s41408-023-00942-3

**Published:** 2023-11-13

**Authors:** Allison M. Bock, Jennifer J. Gile, Melissa C. Larson, Kittika Poonsombudlert, Reema K. Tawfiq, Seth Maliske, Matthew J. Maurer, Brian F. Kabat, Jonas Paludo, David J. Inwards, Sabarish Ayyappan, Brian K. Link, Stephen M. Ansell, Thomas M. Habermann, Thomas E. Witzig, Grzegorz S. Nowakowski, James R. Cerhan, Umar Farooq, Yucai Wang

**Affiliations:** 1 Division of Hematology, Department of Medicine, Mayo Clinic, Rochester, MN USA; 2 Division of Hematology and Hematologic Malignancies, Huntsman Cancer Institute/University of Utah, Salt Lake City, UT USA; 3 Department of Quantitative Health Sciences, Mayo Clinic, Rochester, MN USA; 4 Department of Medicine, Mayo Clinic, Rochester, MN USA; 5 Division of Hematology, Oncology, and Bone and Marrow Transplantation, Department of Internal Medicine, University of Iowa, Iowa City, IA USA; 6 Sanford Health System, Fargo, ND USA

**Keywords:** Epidemiology, B-cell lymphoma, Molecularly targeted therapy

## Abstract

Over the last two decades, the frontline therapy for mantle cell lymphoma (MCL) has evolved. However, the impact of subsequent lines of therapy on survival outcomes has not been well characterized. In this study, we investigated the treatment patterns and survival outcomes in patients with relapsed/refractory (R/R) MCL treated with second-line (2 L) therapy. Adult patients with newly diagnosed MCL from 2002 to 2015 were enrolled in a prospective cohort study. Clinical characteristics, 2 L treatment details, and outcomes were compared between patients who received 2 L treatment between 2003–2009 (Era 1), 2010–2014 (Era 2), and 2015–2021 (Era 3). 2 L treatment was heterogenous in all eras, and there was a substantial shift in the pattern of 2 L therapy over time. The estimated 2-year EFS rate was 21% (95% CI, 13–35), 40% (95% CI, 30–53), and 51% (95% CI, 37–68) in Era 1–3 respectively, and the 5-year OS rate was 31% (95% CI, 21–45), 37% (95% CI, 27–50), and 67% (95% CI, 54–83) in Era 1–3, respectively. These results provide real-world evidence on evolving treatment patterns of 2 L therapy based on the era of relapse. The changes in 2 L treatment correlated with improved EFS and OS, suggesting that treatment advances are associated with improved outcomes in patients with R/R MCL.

## Introduction

Mantle cell lymphoma (MCL) is a B-cell lymphoma characterized by the presence of t(11;14) which leads to overexpression of cyclin D1 [1, 2]. Despite this common genetic abnormality, the clinical presentation of MCL is heterogenous, ranging from an indolent (e.g., leukemic non-nodal MCL) to highly aggressive (e.g., blastoid variant) [2–4]. This clinical heterogeneity has led to difficulty in establishing a uniform standard of care. For younger patients and those “fit” enough to tolerate intensive frontline treatment, there has been general consensus that autologous stem cell transplantation (ASCT) should be considered after frontline induction therapy [5, 6] with regimens containing high dose cytarabine (HiDAC) such as Nordic regimen [6] or R-CHOP alternating with R-DHAP [7], though this approach may be challenged by results of the TRIANGLE study evaluating ibrutinib with and without ASCT [8]. For older patients or those ineligible for transplantation, immunochemotherapy without ASCT is preferred. “Older” regimens and R-CHOP have been slowly replaced by rituximab and bendamustine (R-Bendamustine), which showed less toxicity and an improved median progression-free survival (PFS) compared to R-CHOP [9, 10]. While many patients respond well to frontline treatment, only a small proportion of patients achieve a long-term durable remission. Response to subsequent lines has been historically low with shorter survival than other lymphoma subtypes [11, 12].

The improved understanding of biological mechanisms driving MCL has led to the development of targeted therapies and chemotherapy-free regimens for relapsed and/or refractory (R/R) disease. Early targeted therapies including single-agent bortezomib [13], mTOR inhibitors temsiroliumus [14] and everolimus [15, 16], and the immunomodulatory drug lenalidomide [17] have demonstrated modest single-agent activity in MCL. These agents may have an improved toxicity profile compared to historical chemotherapy regimens, but durable remissions are limited. One of the first successful chemotherapy-free combination regimens was lenalidomide and rituximab which had an overall response rate (ORR) of 92% and a 3-year PFS rate of 80% [18]. The first Bruton’s tyrosine kinase inhibitor (BTKi) ibrutinib was approved as a single agent in 2013 after demonstrating a 68% ORR R/R MCL that was heavily pretreated [19]. Newer BTKi approved subsequently include acalabrutinib and zanubrutinib, which appeared to have less toxicity but similar efficacy compared to ibrutinib [20, 21]. Anti-CD19 chemic antigen receptor (CAR) T-cell therapy with brexucabtagene autoleucel has also demonstrated encouraging durable remissions in a subset of patients, even those with blastoid morphology or *TP53* alterations, and has been approved by FDA for R/R MCL [22, 23]. Incorporation of these therapies in practice may have gradually improved the outcomes of R/R MCL over the years.

Over the last two decades, the treatment landscape for MCL has been evolving rapidly. We previously characterized the shift in frontline therapy and the associated improvement in event-free survival (EFS) and overall survival (OS) [24]. However, the changes in treatment and outcomes of R/R MCL have not been well studied. Such data are needed to understand the uptake of new therapies in practice and examine the impact of novel therapies on outcomes in real-world populations where frontline treatment is also evolving. In this study, we investigated the change in treatment patterns and survival outcomes in patients with R/R MCL treated with second-line (2 L) therapy according to the era of receipt of R/R treatment.

## Methods

### Patients

This study was approved by the institutional review boards at Mayo Clinic and the University of Iowa and was conducted in accordance with the declaration of Helsinki. Adult patients with newly diagnosed MCL between August 2002 and April 2015 and followed through 2021 were identified from the Molecular Epidemiology Resource (MER) prospective cohort of the University of Iowa/Mayo Clinic Lymphoma Specialized Program of Research Excellence (SPORE) [25]. Patients who initiated 2 L therapy for R/R MCL were included in this analysis. Clinical characteristics, first-line (1 L) and 2 L therapies, and treatment outcomes were abstracted from MER and medical records. Frontline treatment was classified as HiDAC based, which included R-Hyper-CVAD/R-MA, R-maxi-CHOP/R-HiDAC (Nordic regimen) or R-CHOP/R-DHAP with or without ASCT consolidation, R-CHOP/R-CHOP-like with or without ASCT, R-Bendamustine with or without ASCT, other systemic therapy (included rituximab/cladribine with and without temsirolimus, fludarabine/rituximab or fludarabine/rituximab/mitoxantrone) and non-systemic therapy (surgical resection or radiation). Second-line therapies were classified the same as frontline therapy in addition to induction (any regimen) followed by allogeneic transplant, BTKi (ibrutinib, acalabrutinib, or zanubrutinib), and other (non-BTKi) targeted therapies (included lenalidomide, temsirolimus, bortezomib, ibritumomab, sorafenib, venetoclax, everolimus, single-agent rituximab). Patterns of 2 L treatment in each year were examined (Supplemental Figure 1), and three treatment eras were defined based on the compositions of 2 L treatments and the changes over time: 2003–2009 (Era 1, enriched for other systemic and non-BTKi targeted therapies), 2010–2014 (Era 2, enriched for R-Bendamustine), and 2015–2021 (Era 3, enriched for BTKi).

### Statistical analyses

Treatment responses were assessed by treating physicians. EFS was defined as the time from 2 L initiation to progression, unplanned retreatment, or death due to any cause. OS was defined as the time from 2 L initiation to death due to any cause. EFS and OS were analyzed using the Kaplan-Meier method. A spline plot was generated to visualize the hazard ratio (HR) for EFS and OS over time from the initiation of 2 L treatment. HR and 95% confidence intervals (CI) were calculated using Cox proportional hazard models with comparisons between eras adjusted for gender and simplified MCL International Prognostic Index (sMIPI). The cumulative incidence of lymphoma-related death was estimated and compared between groups (eras) using a Fine-Gray competing-risk regression model, with deaths from other causes as competing risks. Statistical analyses were performed using R (v 4.1.2). A *p* value < 0.05 was considered statistically significant.

## Results

### Patients

Among a total of 343 MCL patients with a median follow-up of 7.6 years, 86 patients were alive without progression, 60 patients died without progression, and 197 patients had disease relapse or progression after 1 L therapy (Supplemental Figure 2). Of those, 183 received 2 L therapy and had complete treatment information available (*n* = 61 in Era 1, *n* = 73 in Era 2, *n* = 49 in Era 3). At the time of 2 L therapy, 131 (72%) patients had an age >60 years, 147 (80%) were male, 142 (89%) had stage III/IV disease, and simplified MIPI was low in 37 (33%), intermediate in 43 (39%), and high in 31 (28%) patients (missing in 72 patients). No statistical differences in age, gender, stage, or simplified MIPI were found among different eras (Table 1). Progression of disease within 24 months (POD24) of 1 L treatment occurred in 72%, 53%, and 27% among patients in Era 1, Era 2, and Era 3, respectively.

**Table 1:** Baseline characteristics of patients at the time of second-line therapy.

Variable	Era1: 2003–2009 (*n* = 61)	Era2: 2010–2014 (*n* = 73)	Era3: 2015–2021 (*n* = 49)	Total (*N* = 183)	*P* value
Age (years)					
18–60	19 (31.1%)	19 (26.0%)	14 (28.6%)	52 (28.4%)	
>60	42 (68.9%)	54 (74.0%)	35 (71.4%)	131 (71.6%)	
Gender					0.925
Female	11 (18.0%)	15 (20.5%)	10 (20.4%)	36 (19.7%)	
Male	50 (82.0%)	58 (79.5%)	39 (79.6%)	147 (80.3%)	
Stage					0.248
I/II	6 (11.3%)	5 (7.4%)	7 (17.9%)	18 (11.2%)	
III/IV	47 (88.7%)	63 (92.6%)	32 (82.1%)	142 (88.8%)	
Missing	8	5	10	23	
ECOG PS					0.05
0–1	44 (86.3%)	53 (86.9%)	40 (100.0%)	137 (90.1%)	
2–4	7 (13.7%)	8 (13.1%)	0 (0.0%)	15 (9.9%)	
Missing	10	12	9	31	
LDH					0.525
Not elevated	35 (87.5%)	44 (88.0%)	23 (79.3%)	102 (85.7%)	
Elevated (>ULN)	5 (12.5%)	6 (12.0%)	6 (20.7%)	17 (14.3%)	
Missing	21	23	20	64	
B symptoms					0.559
No	45 (90%)	57 (85.1%)	31 (79.5%)	133 (85.3%)	
Yes	5 (10.0%)	10 (14.9%)	8 (20.5%)	23 (14.7%)	
Missing	11	6	10	27	
Bone marrow involvement					0.258
No	13 (35.1%)	20 (50.0%)	9 (50.0%)	42 (44.2%)	
Yes	24 (64.9%)	20 (50.0%)	9 (50.0%)	53 (55.8%)	
Missing	24	33	31	88	
Blastoid or pleomorphic morphology					0.028
No	4 (44.4%)	12 (70.6%)	14 (82.4%)	30 (69.8%)	
Yes	5 (55.6%)	5 (29.4%)	3 (17.6%)	13 (30.2%)	
Missing	52	56	32	140	
Simplified MIPI Group					0.739
Low risk (0–3)	15 (38.5%)	12 (27.9%)	10 (34.5%)	37 (33.3%)	
Intermediate risk (4–5)	12 (30.8%)	19 (44.2%)	12 (41.4%)	43 (38.7%)	
High risk (6–11)	12 (30.8%)	12 (27.9%)	7 (24.1%)	31 (27.9%)	
Missing	22	30	20	72	
POD24 to 1L therapy					<0.001
No	17 (28.3%)	34 (46.6%)	36 (73.5%)	87 (47.8%)	
Yes	43 (71.7%)	39 (53.4%)	13 (26.5%)	95 (52.2%)	
Missing	1	0	0	1	
2L stem cell transplant					0.500
Autologous	5 (8.2%)	10 (13.7%)	4 (8.2%)	19 (10.4%)	
Allogeneic	2 (3.3%)	1 (1.4%)	3 (6.1%)	6 (3.3%)	
No transplant	54 (88.5%)	62 (84.9%)	42 (85.7%)	158 (86.3%)	
2L rituximab maintenance (post BR or ASCT)					0.039
Yes	4 (6.6%)	16 (21.9%)	10 (20.4%)	30 (16.4%)	
No	57 (93.4%)	57 (78.1%)	39 (79.6%)	153 (83.6%)	

*1L* first-line, *2L* second-line, *ASCT* autologous stem cell transplant, *BR* bendamustine-rituximab, *ECOG PS* eastern cooperative oncology group performance status, *LDH* lactate dehydrogenase, *MIPI* Mantle Cell Lymphoma international prognostic index, *POD24* progression of disease within 24 months, *ULN* upper limit of normal.

### Treatment patterns

**Fig. 1:**
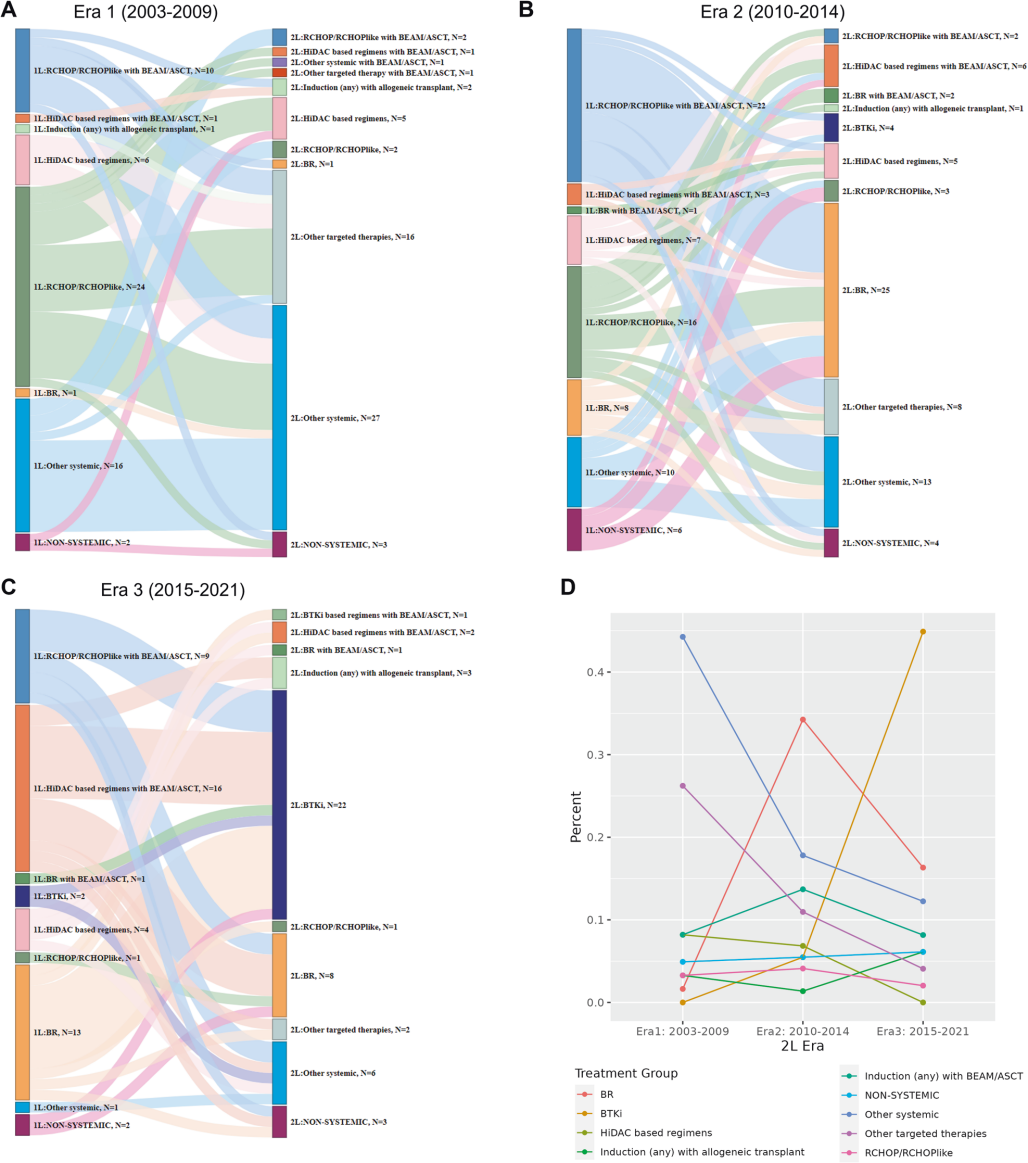
Patterns of 2 L treatment and prior 1 L treatment by era of 2 L treatment. **A**–**C** Patterns of treatment in Era 1–3. **D** Summary of treatment groups by era of 2 L treatment. Abbreviations: 1 L first-line, 2 L second line, ASCT autologous stem cell transplant, BEAM carmustine, etoposide, cytarabine, and melphalan, BR, bendamustine plus rituximab, BTKi Bruton tyrosine kinase inhibitor, CHOP cyclophosphamide, doxorubicin, vincristine, and prednisone, HiDAC high dose cytarabine, R: rituximab. Treatment groups: HiDAC-based: R-Hyper-CVAD/R-MA, R-maxi-CHOP/R-HiDAC (Nordic regimen) or R-CHOP/R-DHAP; R-CHOP/R-CHOP-like: R-CHOP, R-CVP, R-EPOCH, R-CHOP + methotrexate, R-CHOP + ibritumomab; Other systemic therapy: rituximab/cladribine with and without temsirolimus, fludarabine/rituximab, cladribine/fludarabine or fludarabine/rituximab/cyclophosphamide/mitoxantrone, VcR-CVAD; Targeted therapy: lenalidomide with or without rituximab, bortezomib, ibritumomab, sorafenib, venetoclax, everolimus, temsirolimus, single agent rituximab; BTKi: ibrutinib, acalabrutinib, or zanubrutinib; Non-systemic therapy: surgical resection (including splenectomy) or radiation therapy.

There were substantial heterogeneities in both 2 L and 1 L treatments across all eras. The patterns of 2 L treatment by era, and previous 1 L treatment, are shown in Fig. 1A–C. Notable changes in 2 L treatment patterns were observed among different eras, likely related to 2 L drug availability and 1 L treatment choices. In Era 1, the common 2 L choices were other (non-BTKi) targeted therapies (*n* = 16, 26%) or other systemic therapies (*n* = 26, 43%) which mainly include rituximab and single-agent chemotherapy combinations. In Era 2, common choices were R-Bendamustine (*n* = 25, 34%), followed by other systemic (*n* = 13, 18%) and other (non-BTKi) targeted therapies (*n* = 8, 11%). In Era 3, BTKi was the most common 2 L therapy (*n* = 23, 47%) followed by other systemic therapies (*n* = 6, 12.2%). The use of other (non-BTKi) targeted therapies was highest in Era 1 (*n* = 17; 28%) compared to Era 2 (*n* = 8; 11%) and Era 3 (*n* = 2; 4%), reflecting a higher use of lenalidomide, mTOR inhibitors such as everolimus, and R-cladribine at relapse in Era 1. Few to no patients received a BTKi in 2 L in Era 1 (*n* = 0) and Era 2 (*n* = 4; 6%), largely due to lack of availability, in contrast to Era 3 (*n* = 23, 47%). The use of R-Bendamustine in 2 L was minimal in Era 1 (*n* = 1; 2%) and less in Era 3 (*n* = 9; 18%) compared to Era 2 (*n* = 27; 37%), reflecting the impact of BTKi availability in Era 3 on 2 L treatment choice. HiDAC-containing regimens in 2 L was higher in Era 1 (*n* = 6; 10%) and Era 2 (*n* = 11; 15%) compared to Era 3 (*n* = 2, 4%). The percentage of patients who received autologous or allogeneic stem cell transplants in the 2 L appeared similar: Era 1 (*n* = 7; 12%), Era 2 (*n* = 11; 15%), and Era 3 (*n* = 7; 14%). The use of rituximab maintenance in 2 L was similar in Era 2 (*n* = 16; 22%) and Era 3 (*n* = 10; 20%) but higher than Era 1 (*n* = 4; 7%). The change in frequency of each treatment group in different eras is shown in Fig. 1D. More detailed view of these changes by year of 2 L treatment is shown in Supplemental Figure 1.

### Treatment response and survival outcomes

**Fig. 2:**
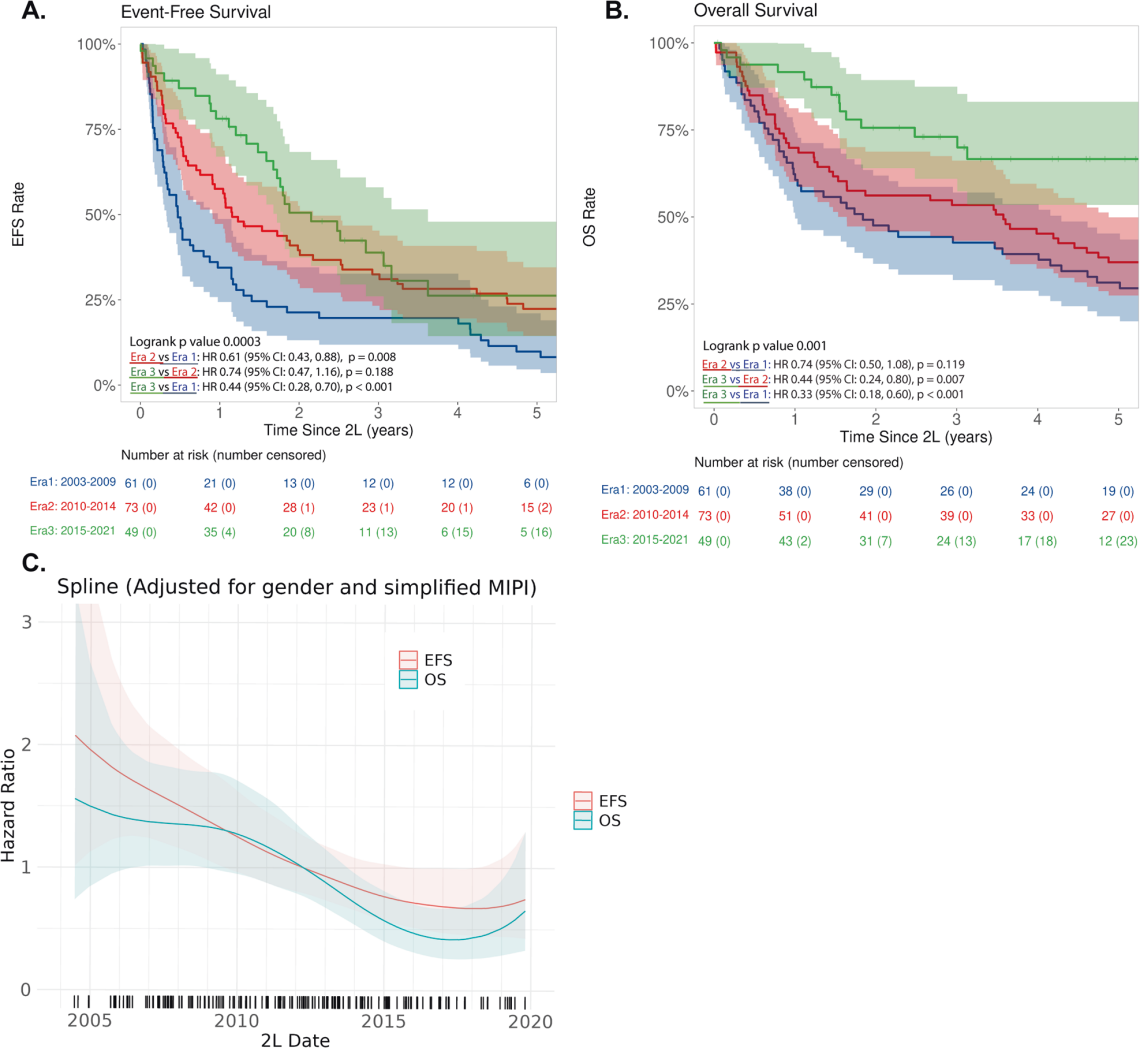
EFS and OS from 2 L treatment in three different Eras. **A** EFS in Eras 1–3. **B** OS in Eras 1–3. **C** Spline plot of EFS and OS over time adjusted for gender and simplified MIPI.

The median follow-up from 2 L therapy in Eras 1–3 was 12.4, 8.7, and 4.2 years, respectively. The overall response rate to 2 L therapy in Eras 1–3 was 56% (95% CI: 42–69), 80% (95% CI: 68–89), and 88% (95% CI: 72–95), respectively. The complete response rate was 31% (95% CI: 20–45), 54% (95% CI: 41–66) and 53% (95% CI: 36–68), respectively (Table 2). EFS and OS improved over time (Fig. 2A, B). The estimated 2-year EFS rate was 21% (95% CI: 13–35) in Era 1, 40% (95% CI: 30–53) in Era 2, and 51% (95% CI: 37–68) in Era 3 (*p* < 0.001, Fig. 2A and Table 2). The estimated 5-year OS rate was 31% (95% CI: 21–45) in Era 1, 37% (95% CI: 27–50) in Era 2, and 67% (95% CI: 54–83) in Era 3 (*p* < 0.001, Fig. 2B and Table 2).

**Table 2:** Outcomes of second-line therapy in patients with mantle cell lymphoma by era of second-line therapy.

	Era 1 2003–2009 (*n* = 61)	Era 2 2010–2014 (*n* = 73)	Era 3 2015–2021 (*n* = 49)	Total (*N* = 183)
ORR (95% CI)	56% (42–69)	80% (68–89)	88% (72–95)	73% (66–80)
CR (95% CI)	31% (20–45)	54% (41–66)	53% (36–68)	45% (38–53)
2–year EFS (95% CI)	21% (13–35)	40% (30–53)	51% (37–68)	36% (30–44)
Median EFS (years)	0.5	1.2	2.2	1.2
5–year OS (95% CI)	31% (21–45)	37% (27–50)	67% (54–83)	41% (34–49)
Median OS (years)	1.8	3.6	NR	3.6

*CI* confidence interval, *CR* complete response, *EFS* event-free survival, *NR* not reached, *ORR* overall response rate, *OS* overall survival.

**Fig. 3:**
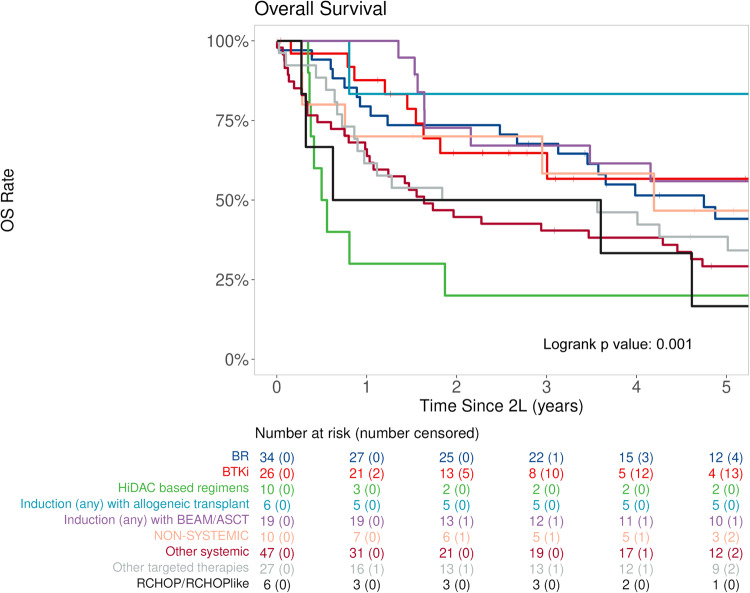
Overall survival by second-line therapy in patients with relapsed mantle cell lymphoma. Abbreviations/treatment groups: BR bendamustine plus rituximab, BTKi Bruton tyrosine kinase inhibitor, HiDAC-based R-Hyper-CVAD/R-MA, R-maxi-CHOP/R-HiDAC (Nordic regimen) or R-CHOP/R-DHAP, R-CHOP/R-CHOP-like R-CHOP, R-CVP, R-EPOCH, R-CHOP + methotrexate, R-CHOP + ibritumomab, ASCT autologous stem cell transplant, BEAM carmustine, etoposide, cytarabine, and melphalan, Other systemic therapy rituximab/cladribine with and without temsirolimus, fludarabine/rituximab, cladribine/fludarabine or fludarabine/rituximab/cyclophosphamide/mitoxantrone, VcR-CVAD, Other targeted therapies lenalidomide with or without rituximab, bortezomib, ibritumomab, sorafenib, venetoclax, everolimus, temsirolimus, single-agent rituximab, BTKi ibrutinib, acalabrutinib, or zanubrutinib, Non-systemic therapy surgical resection (including splenectomy) or radiation therapy, CHOP cyclophosphamide, doxorubicin, vincristine, and prednisone, R-CHOP/R-CHOP-like R-CHOP, R-CVP, R-EPOCH, R-CHOP + methotrexate, R-CHOP + ibritumomab.

The spline plots depict a trend in decreased hazard in EFS and OS over time (Fig. 2C). For example, compared to 2 L start in April 2012 (median 2 L start date, reference point), 2 L start in January 2008 and January 2017 had an HR for EFS of 1.5 (95% CI: 1.2–2.0) and 0.7 (95% CI: 0.5–1.0) respectively, and an HR for OS of 1.4 (95% CI: 1.0–1.8) and 0.4 (95% CI: 0.3–0.7), respectively. Overall survival based on 2 L treatment choice is shown in Fig. 3. There was a trend towards improved OS with BTKi and R-Bendamustine compared with other systemic therapies, suggesting the benefits of newer therapy options. In addition, autologous and allogeneic stem cell transplant was associated with favorable outcomes as well, suggesting the feasibility of transplant for R/R MCL in select patients.

**Fig. 4:**
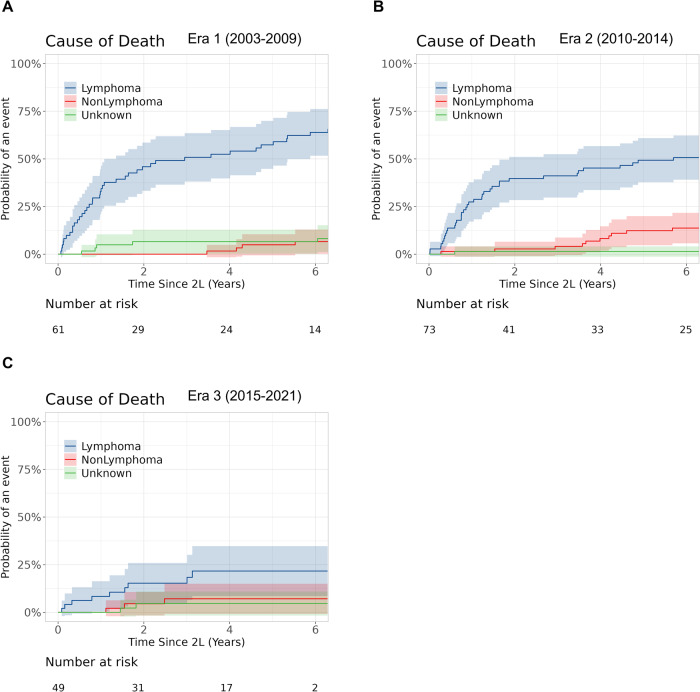
Cumulative incidence of lymphoma-related death by Era. **A**–**C** Cumulative incidence of lymphoma related death compared to non-lymphoma-related death and death from unknown causes in Eras 1–3.

The cumulative incidence of lymphoma-related death at 2 years was 45.9% (95% CI: 34.8–60.5), 39.7% (95% CI: 29.9–52.8), and 15.3% (95% CI: 7.6–30.6) in Era 1–3, respectively, and at 5-years was 57.4% (95% CI: 46.1–71.5), 49.3% (95% CI: 39.0–62.4), and 21.7% (95% CI: 11.8–39.7), respectively (Fine-Gray *p* value < 0.001)(Fig. 4). Among patients who died (*n* = 124), the proportion of disease progression related death declined over time and was 68%, 59%, and 50% in Eras 1–3, respectively (Table 3).

**Table 3:** Primary cause of death by era of second-line therapy.

Primary cause of death	Era1 2003–2009 (*n* = 56)	Era2 2010–2014 (*n* = 54)	Era3 2015–2021 (*n* = 14)	Total (*N* = 124)
Disease progression	38 (67.9%)	32 (59.3%)	7 (50.0%)	77 (62.1%)
Therapy-related, infection	3 (5.4%)	6 (11.1%)	2 (14.3%)	11 (8.9%)
Therapy-related, other	2 (3.6%)	1 (1.9%)	0 (0.0%)	3 (2.4%)
Second malignancy	4 (7.1%)	4 (7.4%)	2 (14.3%)	10 (8.1%)
Other causes, unrelated to lymphoma	2 (3.6%)	9 (16.7%)	1 (7.1%)	12 (9.7%)
Unknown (unable to obtain records)	7 (12.5%)	2 (3.7%)	2 (14.3%)	11 (8.9%)

## Discussion

In this study, we found that 2 L therapy for MCL evolved with time and was likely affected by 1 L treatment choice and the availability of treatment options at the time of relapse. While there has been a shift in the pattern of treatment, both 1 L and 2 L treatment choices across each era remain heterogeneous. Our data also demonstrate that a change in treatment patterns at the time of first relapse is associated with improved post-relapse outcomes. The improvement in EFS and OS is seen in both Era 2 and Era 3, emphasizing the impact of many recently approved therapies on improving survival outcomes for R/R MCL.

Several population-based studies have examined MCL outcomes and found improved overall survival with time, though the analysis in many was focused on the impact of frontline treatment or was limited by the heterogeneous use of rituximab in frontline immunochemotherapy which has been well documented to have a survival benefit [12, 26, 27]. Smith et al. reported improved outcomes in R/R MCL in the U.K. population but only 30% of patients received rituximab with frontline chemotherapy prior to 2015 and only 9% of patients received consolidation ASCT, both of which are associated with improved outcomes in other studies [27]. The largest R/R MCL analysis to our knowledge reported outcomes of a primarily community-based population and had robust data on BTKi utilization, but was limited by a low use of cytarabine-based induction and consolidative ASCT in younger patients which may impact the interpretation of treatment patterns and outcomes in the younger population [28]. Although MCL has historically had a poor prognosis, our data support and complement other studies that have reported an improvement in overall survival over time [27], and importantly, we demonstrate the benefit of novel therapies at relapse in a population that received upfront immunochemotherapy with high rates of consolidation ASCT in younger patients. Additionally, our study captures the impact of newer BTKi generations such as acalabrutinib which were approved after 2015. The improvement in outcomes since 2015 may reflect the durable benefit of ibrutinib and newer generations of BTKi. While the methodology of this study precludes any firm attribution of improved survival outcomes to specific therapies, in aggregate the findings suggest the success of the current regulatory strategies for approval of novel agents with apparent impact on surrogate survival endpoints in phase II clinical trials [19–22].

There is no consensus on preferred 2 L therapy at the time of relapse, with treatment selection impacted by patient factors, frontline therapy, duration of remission, and ability to undergo autologous or allogeneic stem cell transplant. Several known biological tumor features also influence treatment selection including high Ki-67 index, *TP53* alterations (deletion of chromosome 17p or mutations in *TP53* gene), and the presence of blastoid morphology [1]. Our findings may reflect the diversity in MCL presentation and biologic features, which can lead to different patterns of care in both 1 L and 2 L. Combination strategies of BTKi with other targeted therapies (e.g., venetoclax, rituximab) aimed at improving depth of response are currently being evaluated. There are numerous single agent and combination treatments with targeted therapies currently approved or being tested in clinical trials including venetoclax and CD20xCD3 bispecific antibodies [29]. With multiple treatment options available, the optimal treatment and sequence remains to be established and the treatment patterns will continue to evolve.

The strengths of this study include the prospective cohort study design, availability of detailed treatment information at relapse, and length of follow-up. We have previously published outcomes for frontline treatment before and after 2010 [24]. This study provides valuable information on the impact of therapies at relapse on event-free and overall survival outcomes. Of note, both 1 L and 2 L treatments were heterogeneous across all eras, and it is unclear whether the baseline characteristics and the treatment heterogeneities in 1 L would have affected the response and prognosis in 2 L. Nevertheless, both our frontline [24] and R/R studies do clearly demonstrate the improving MCL outcomes in the evolving treatment landscape. Limitations include lack of racial diversity, and lack of consistent documentation of high-risk features such as blastoid morphology or *TP53* alterations. While venetoclax is a promising newer therapy, we had a very small number of patients treated with venetoclax during Era 3 (*n* = 2) and very few patients who underwent CAR T-cell therapy (*n* = 3, all beyond 2 L), so our study is not positioned to draw conclusions on the benefit of these and other new therapies. The sample size and follow-up also limited us to further analyze third-line and beyond treatments for R/R MCL.

In summary, we found that survival outcomes for R/R MCL have improved over time with the introduction of novel therapies into clinical practice. Our study highlights the impact of treatment changes in real-world settings. Our outcomes for R/R MCL are improved compared to historical studies and provide valuable information for future clinical trial design (e.g., benchmarks for efficacy outcomes in single-arm trials, appropriate control arm considerations given the heterogeneity, etc). This is especially important as MCL is an uncommon lymphoma and large trials are challenging to conduct with the rapidly changing treatment landscape.

### Supplementary information

Supplemental material

AJ checklist

## Data Availability

The datasets generated during and/or analyzed during the current study are available on request from the corresponding author on reasonable request. The data are not publicly available due to privacy or ethical restrictions.
