# Supplementary material for: Evolving treatment patterns and improved outcomes in relapsed/refractory mantle cell lymphoma: a prospective cohort study
Source: Blood Cancer J. 2023 Nov 13;13(1):169. doi: 10.1038/s41408-023-00942-3 (PMC10643454; doi:10.1038/s41408-023-00942-3)
Supplement: Supplementary file 1 — Supplemental material [file 41408_2023_942_MOESM1_ESM.docx]

**Supplementary Figure 1. Second-line treatment changes by year and era**

The vertical dotted lines indicate boundaries of eras.


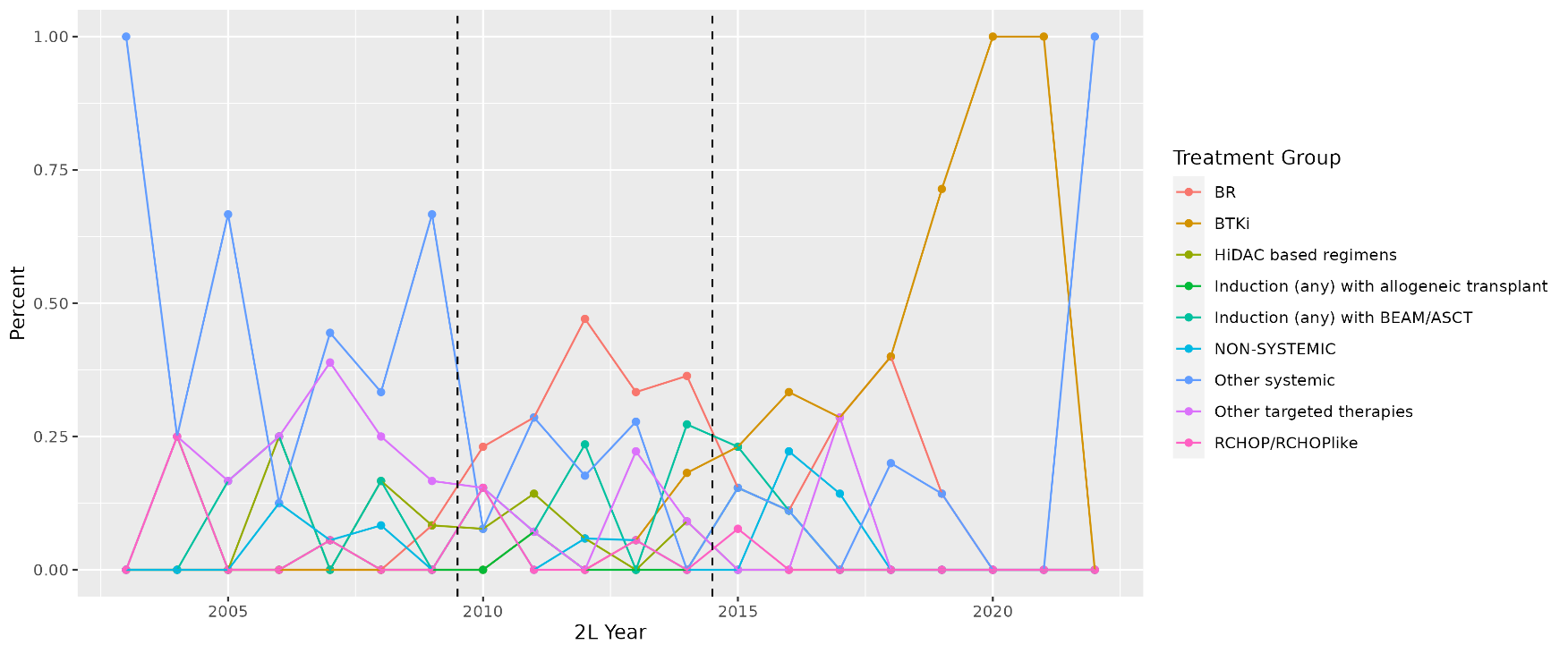


**Supplementary Figure 2. Consort diagram of patient distribution**

Assessed for eligibility (N=343)

Excluded (n=160)

- Alive without progression (n=86)
- Died without progression (n=60)
- Relapsed with incomplete treatment data (n=14)

Received second-line therapy (n=183)

Era 2 (2010-2014)

n=73

Era 3 (2015-2021)

n=49

Era 1 (2003-2009)

n=61
